# HIV-Tat and vascular endothelium: implications in the HIV associated brain, heart, and lung complications

**DOI:** 10.3389/fimmu.2025.1621338

**Published:** 2025-08-07

**Authors:** Sivasankar Chandran, Morgan Adler, Ling Chen, Sandeep Kaur, Navneet K. Dhillon

**Affiliations:** Division of Pulmonary, Critical Care and Sleep Medicine, Department of Internal Medicine, University of Kansas Medical Center, Kansas City, KS, United States

**Keywords:** endothelium, Tat, blood brain barrier, pulmonary vascular remodeling, cardiovascular dysfunction

## Abstract

Following the advent of antiretroviral therapy (ART), neurological, cardiovascular, and pulmonary comorbidities emerged as major challenges in treating non-infectious complications in people living with HIV. Despite effective ART, HIV viral proteins can persist in circulation even in individuals with negligible viral loads, potentially contributing to cellular and tissue-level stress, inflammation, and related health complications. Most of the HIV protein: Tat (Trans activator of Transcription), expressed in HIV-infected cells, is actively secreted and exerts its pathological effects on non-infected cells, particularly impacting the vascular endothelium. This review focuses on the role and the underlying mechanisms of HIV-Tat in promoting endothelial dysfunction across the cardiovascular, pulmonary, and brain vasculature. Additionally, we discuss how HIV-Tat interacts synergistically with drugs of abuse to exacerbate endothelial damage. Importantly, the vascular damage caused by Tat is not fully mitigated by HAART, necessitating further mechanistic investigations and targeted therapeutic interventions. Additionally, cessation of drug abuse is indispensable for improving clinical outcomes and restoring vascular health in people living with HIV.

## Introduction

According to WHO, 36.1-44.6 million patients were surviving with HIV at the end of 2023, with approximately 1.3 million new infections occurring that year. Advancements in Human immunodeficiency virus (HIV) care and treatment have transformed the infection into a manageable chronic condition. Consequently, with access to and adherence to antiretroviral therapy (ART), people living with HIV (PLWH) can now attain a life expectancy comparable to that of the general population worldwide (1). However, HIV is frequently associated with comorbid conditions, particularly cardiopulmonary and neurocognitive disorders.

The incidence of cardiovascular diseases (CVD), such as coronary artery disease and myocardial infarctions, has tripled over the past two decades, making them a leading cause of hospitalization, disability, and mortality among PLWH (2–4). In addition, HIV significantly increases the risk of obstructive lung disease and pulmonary vascular complications (5). Among pulmonary complications, pulmonary arterial hypertension (PAH) is one of the most severe, with a high mortality rate (6). PAH is more prevalent among PLWH than those without HIV, and its overall prevalence has remained largely unchanged since the introduction of ART. The relationship between ART and PAH severity remains inconclusive, as disease severity does not consistently correlate with ART use (7). Additionally, HIV exacerbates pulmonary hypertension (PH) in PLWH with left heart disease (PH-LHD) by increasing right ventricular systolic pressure (RVSP) and reducing survival rates. The elevated RVSP and lower body mass index (BMI) remain significant predictors of PH-LHD mortality (8). Studies have shown varying prevalence rates of PAH among HIV-infected cohorts, ranging from 0.46% to 0.5% in larger populations before and after Highly Active Anti-retroviral Therapy (HAART) introduction (9–11). Studies using echocardiography and different Pulmonary Artery Systolic Pressure (PASP) thresholds reported higher rates of PH (PH), from 2.6% up to 9.9% (12–14). In recent study based on US national inpatient data, 3.19% of hospitalized PLWH were identified with pulmonary hypertension (PH). Compared to those with HIV alone, patients with both HIV and PH had significantly burden of comorbidities including heart failure, cardiogenic shock cardiomyopathy, cardiac arrest and respiratory failure (15).

HIV infection is also linked to a rising incidence of cerebrovascular diseases, with increasing hospitalizations over time. HIV-associated neurological complications heighten stroke risk, leading to higher mortality, morbidity, disability, and a greater likelihood of long-term care facility discharge (16). Additionally, major depressive disorder (MDD) is two to four times more common in PLWH than in the general population. Estimates of HIV-associated neurocognitive disorder (HAND) range from 25% to over 47% among PLWH (17). Although combination ART (cART) has significantly reduced the prevalence of severe forms of HAND, such as HIV-associated dementia, mild and moderate neurocognitive impairments continue to persist (18). This could be attributed to the persistence of chronic inflammation in PLWH on ART, with 20-25% developing severe inflammation after treatment (19). This inflammation is partly due to low-level transcription of HIV genes, encoding early HIV proteins such as Tat (Trans activator of transcription), Rev (Regulator of virion), and Nef (Negative regulatory factor). These viral proteins have profound implications beyond their virological roles, particularly HIV-Tat, which has been associated with multiple HIV-associated comorbidities (20). Endothelial dysfunction has been identified as a significant contributor to numerous HIV-associated comorbidities (21). HIV proteins, including gp120, Nef, and Tat, are involved in inducing endothelial dysfunction (22–26). This review highlights the critical role of HIV-Tat in disrupting endothelial function and its downstream implications in brain, heart, and lung-associated complications in PLWH.

## HIV-Tat characteristics

HIV-Tat is a non-structural, regulatory protein of HIV-1, with a molecular weight ranging from 14 to 16 kDa. HIV-Tat binding to the HIV-LTR (long terminal repeat) promoter using another viral RNA element, TAR (transactivation responsive region), plays a crucial role in the viral life cycle by enhancing transcription, particularly transcript elongation (27). Notably, Tat is well recognized for its function in releasing RNA polymerase II from its paused state, thereby facilitating elongation, a critical step in the completion of HIV gene transcription (28). Additionally, Tat also plays an essential function in initiating reverse transcription, accelerating transcription rates (29), and participating in the regulation of splicing (30). Further, Tat can directly bind to the Nuclear Factor kappa B (NF-κB) enhancer sequence in the LTR, enabling TAR-independent transactivation of the HIV-1 LTR (31). A recent study revealed that Tat activates the NF-κB pathway through a direct interaction with Tumor Necrosis Factor Receptor-Associated Receptor 6 (TRAF6). This interaction promotes TRAF6 oligomerization and ubiquitination, resulting in NF-κB activation and HIV-LTR transactivation. This mechanism, conserved across HIV-1, HIV-2, and Simian Immunodeficiency Virus (SIV), highlights the importance of TRAF6 as a key regulator of viral gene expression (32). SP1 transcription factor can also potentiate Tat-mediated transactivation of HIV-LTR independent of TAR (31).

**Figure 1:**
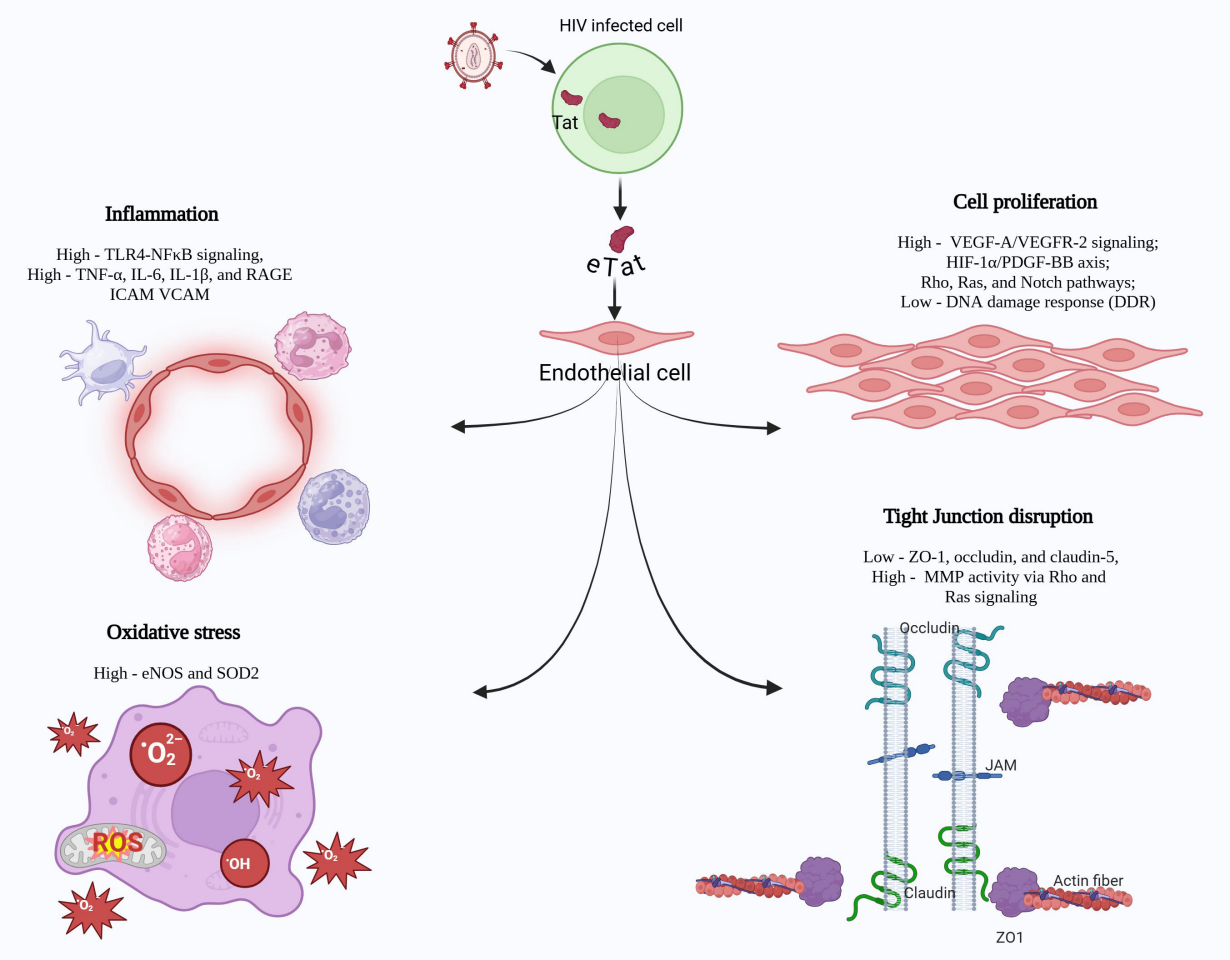
HIV-Tat, expressed by actively or latently infected macrophages and T cells, is secreted into the extracellular environment and damages neighboring or far-off non-infected cells, leading to range of pathological molecular alterations. These mainly include 1) heightened pro-inflammatory signaling, characterized by upregulated TLR4/NFκB activity, 2) enhanced oxidative stress, primarily through the dysregulation of eNOS and SOD2 expression, 3) hyperproliferation of vascular endothelial cells by modulating the VEGF, PDGF-BB, Rho/Ras or Notch signaling pathways and 4) disruption of tight junction proteins in the endothelium.

The first two domains of Tat are proline-rich and cysteine-rich, contributing to structural stability. According to earlier studies, the third domain engages with tubulin and microtubules through its interaction with the microtubule-associated protein LIS1 (33), resulting in disrupted microtubule dynamics and triggering a mitochondria-dependent apoptotic pathway (34). The fourth and fifth domains are arginine-rich and glutamine-rich, respectively, and play a role in RNA binding. Additionally, sixth region, located in exon 2 at the C terminal, contains an arginine-glycine-aspartic acid (RGD) motif (78–80 aa), which is critical for Tat’s interaction with integrins. This interaction facilitates optimal viral replication in T cells and macrophages (35). A significant portion (nearly 65%) of the Tat protein synthesized in the infected cell is released extracellularly, primarily through a leaderless secretory pathway, without any cell death or alteration in the membrane permeability (36). Tat can traffic through the plasma membrane independently of intracellular intermediates. Specifically, the conserved RKK motif (Arg49, Lys50, and Lys51) of Tat bind to phosphatidylinositol-(4,5)-bisphosphate [PI(4,5)P2] of the plasma membrane (37). This interaction may influence various biological processes involving PI(4,5)P2, including clathrin-mediated endocytosis (38), phagocytosis (39), and other cellular functions. Once bound to the plasma membrane, Tat is subsequently released extracellularly via exocytosis (40). Recent findings suggest that Tat is also secreted extracellularly through extracellular vesicles, which are notably enriched with small noncoding RNAs containing transactivating response (TAR) elements and their derivatives, thus leading to inflammation (41). Importantly, extracellular Tat (eTat) has been shown to traverse the blood-brain barrier, contributing to CNS (central nervous system) inflammation and T-cell activation (42). The concentration of circulating Tat in the bloodstream is estimated to range from 2 ng/mL to 40 ng/mL (43). In individuals with HIV undergoing combination antiretroviral therapy (cART), HIV-Tat was detected in the serum of 25% of patients. Notably, Tat levels in the serum were not influenced by immune suppression or HIV replication status. Persistent secretion of Tat (**Figure 1**) may contribute to the development of HIV-associated complications (44).

## HIV-Tat, inflammation, and endothelial dysfunction

The vascular endothelium plays a fundamental role in maintaining vascular homeostasis. It regulates blood flow, vascular tone, coagulation, leukocyte trafficking, and permeability, ensuring the proper function of organ systems. Endothelial cells (ECs) form a single-cell monolayer lining the blood vessels and are crucial for sensing and responding to physical and chemical signals. Disruption of endothelial function is a hallmark of several diseases, including cardiovascular dysfunction (CVD), neurovascular disorders, and pulmonary pathologies. Dysfunctional endothelium is characterized by oxidative stress, inflammation, increased permeability, and impaired vasorelaxation, leading to tissue injury and organ dysfunction (45).

A study by Dysangco et al. reported that HIV-infected patients, especially those not on ART, had elevated levels of endothelial activation biomarkers such as soluble Vascular Cell Adhesion Molecule 1 (VCAM-1), Tissue inhibitor of metalloproteinases-1, and soluble CD163 when compared to the uninfected control group (46). A study conducted in South Africa on youths receiving ART also revealed impaired endothelial function, as measured through the reactive hyperemic index, when compared to age- and sex-matched HIV-negative controls. Notably, endothelial dysfunction persisted even after 24 months of ART, despite achieving viral suppression (47). Additionally, research indicates that arterial stiffness remains elevated regardless of ART use in PLWH, suggesting that ART alone is insufficient in restoring endothelial function to a healthy state (48, 49).

Tat was the first HIV-1 protein proven to rigorously affect endothelial cells in the pre-ART era, promoting vascular endothelial dysfunction and the inception and progression of angio-proliferative Kaposi sarcoma. Tat released by HIV-infected cells acts on endothelial cells in a paracrine fashion, causing damage to capillaries, increasing permeability, and promoting overexpression of cell adhesion molecules on the endothelial surface. HIV–Tat protein has been shown to promote the cell surface expression of Endothelial leucocyte adhesion molecule-1 (ELAM-1), VCAM-1, and Intercellular Adhesion Molecule-1 (ICAM-1) in human umbilical vein endothelial cells which was associated with enhanced adhesion of monocytes to the endothelial cells (50) in turn, contributing to endothelial activation and vascular inflammation (**Figure 1**) (51, 52). Additionally, the Tat protein can bind lymphocytes having heparan sulfate proteoglycans on their surface. Once bound, Tat can facilitate the adhesion of these lymphocytes to the endothelial cell surface and promote migration of lymphocytes across the endothelium (53), thus suggesting the contribution of Tat to the destruction of tissue parenchyma in PLWH.

HIV-Tat potently mediates oxidative stress and activates nuclear factor-κB (NF-κB), resulting in the over-expression of adhesion molecules such as ICAM-1 VCAM-1, and E-selectin on endothelial cells (54–56). Tat’s ability to activate the NF-κB pathway by either directly binding to NF-κB enhancer sequences (31) or via activation of TRAF6 (32) potentially enables (TAR)-independent transactivation of the cellular gene expression in response to Tat. These interactions reveal novel mechanisms behind Tat’s broad regulatory roles in both viral and host gene expression. Additionally, it has been demonstrated that Tat protein interactions with toll-like receptor 4 (TLR4) on monocytes and its downstream involvement of Myd88 and TRIF pathways, results in PKC, MAP kinase, and NF-κB activation, consequently leading to immune dysregulation via induction of TNFα and IL-10 expression (57). Another study by Nicoli et al. demonstrated that HIV-Tat contributes to immune dysfunction via T-cell hyperactivation and impaired antiviral response (58). CD4 T cells exposed to HIV-Tat demonstrate increased senescence and decreased cell proliferation, further contributing to immune dysfunction (59). This chronic immune dysregulation could result in endothelial activation during cross-talk between circulating and perivascular immune cells and endothelial cells. Tat also induces the production of pro-inflammatory cytokines, including IL-1β, IL-8, IL-6, and MCP-1 in vascular endothelial cells, which attract more inflammatory cells to the vasculature (**Figure 1**) (56, 60–63). Importantly, HIV-1 Tat protein exploits integrins to enter endothelial cells activated by inflammatory cytokines and thereby makes them susceptible to productive virus replication (64). Reports suggest that endothelial cells are specifically exposed to higher concentrations of Tat, locally secreted by macrophages (43, 65). *In vivo* experiments have demonstrated that subcutaneous injection of HIV- Tat protein in mice causes a dose-dependent increase in vascular permeability, promoting infiltration of lymphomononuclear cells with MCP-1 and PAF (Platelet-Activating Factor) playing significant roles in this process (66).

Overall, the disruption of endothelial function by HIV-Tat has systemic consequences, particularly in the brain, heart, and lungs. Through mechanisms including oxidative stress, and inflammation, Tat contributes to severe complications such as neurovascular injury (67), atherosclerosis (68), myocardial dysfunction (69), and pulmonary vascular remodeling (70), as explained in the next sections.

## HIV-Tat and brain vascular injury

Individuals infected with HIV may develop HIV-associated neurocognitive disorders (HAND), which range from asymptomatic neurocognitive impairment to more severe HIV-associated dementia. The development of HAND is linked to the migration of blood-borne monocytes into the central nervous system (CNS) parenchyma across the blood-brain barrier (BBB) (51, 71). This barrier primarily consists of brain endothelial cells that form tight junctions and interact with astrocytes and pericytes, maintaining a protective barrier against blood-borne elements, including inflammatory cells (72). The HIV load in the CNS doesn’t always correlate with the degree of neurologic impairment. Hence, it is proposed that soluble mediators such as Tat play a significant role in the progression of CNS disease (73). A substantial amount of HIV-Tat mRNA and protein was found in the central nervous systems of PLWH with neurodegenerative disease (74, 75). Furthermore, chronic expression of Tat in Tat transgenic mice has been reported to contribute to age-associated comorbidities, such as heightened anxiety-like behavior, cognitive impairment, and enhanced sensitivity to mechanical allodynia compared to Tat-negative aged-matched mice (76). Similar results were reported by Zhao et al., indicating that sustained expression of Tat in aged mice results in both short- and long-term memory deficits, reduced motor activity, impairment in balance and coordination, heightened astrocyte activation, disrupted neuronal integrity, and a reduction in overall genomic DNA methylation (77).

Multiple studies have found that elevated levels of BBB permeability and vascular leakage are a frequent occurrence in the brain tissues of HIV-infected patients (78, 79). Tat can cross the BBB through a mechanism that supports unidirectional influx with a rate of about 0.490 microl/g/min (80). HIV-Tat expressing transgenic mice also had compromised BBB integrity, which was associated with the accumulation of activated phagocytic perivascular macrophages and microglia in the brain (81). Another study reported heightened oxidative stress leading to increased expression of MCP-1in Tat-exposed human brain microvascular endothelial cells (HBMECs) (82) and in brain tissues from mice injected with Tat into the right hippocampus (51). Further, Tat treatment increases expression of E-selectin, CCL-2, and IL-6 in HBMECs (83). This may allow the infiltration of monocytes and HIV-infected cells into the CNS, triggering neuroinflammation and contributing to HIV-associated neurocognitive disorders.

Tight junctions (TJs) are essential for BBB function. Tat protein disrupts the expression and localization of TJ proteins such as ZO-1, occludin, and claudin-5, compromising the BBB integrity (67, 84, 85). Tat also interacts with VEGFR2 and activates Rho-kinase signaling, which results in cytoskeletal reorganization and the disassembly of tight junctions (**Figure 1**) (86). In brain endothelial cells, exposure to Tat not only leads to transcriptional repression but also induces nuclear localization of ZO-1 through Rho signaling and CREB (Cyclic AMP Response Element-Binding Protein) activation. Depleting CREB has been shown to protect against Tat-induced changes in ZO-1 levels and the disruption of endothelial integrity (86).

Moreover, Tat increases cell adhesion to the BBB and has been shown to induce the overexpression of ICAM-1 in HBMECs and microvessels from the mouse brain. Interestingly, the PPARγ (Peroxisome Proliferator-Activated Receptor) agonist was protective against this inflammatory response (52). Another study reported mitigation in Tat-mediated activation of NFκB and increased IL-1β, TNF-α, CCL2, and E-selectin levels in HBMECs overexpressing PPARγ/PPARα or in the presence of PPARγ agonist (87). Exposure of HBMECs to HIV-Tat triggers endoplasmic reticulum stress, marked by activation of key regulators like Glucose-Regulated Protein-78 is a chaperone (GRP78), Activating Transcription Factor 6 (ATF6), and Protein Kinase R-like ER Kinase (PERK), leading to apoptosis and reduced cell viability (88). In this study, Tat was also reported to induce mitochondrial dysfunction as indicated by reduced Bcl2/Bax ratio, increased release of cytochrome c, and loss of mitochondrial potential. Thus, both endoplasmic reticulum stress and mitochondrial dysfunction were highlighted as key drivers of Tat-induced cell death of brain microvascular endothelial cells (88).

Tat also aggravates amyloid-β accumulation, a hallmark of neurodegenerative pathology (89). Injection of Tat in transgenic mice expressing human amyloid precursor protein and Presenilin resulted in increased disruption of ZO-1 tight junction proteins, augmentation in Matrix Metalloproteinase-9 (MMP-9) expression, and enhanced BBB permeability that correlated with amyloid β accumulation (90). Further exposures of mice to Tat protein led to increased BBB permeability accompanied by upregulated expression of RAGE (Receptor for Advanced Glycation End Products) and downregulated expression of LRP1 (Low-Density Lipoprotein Receptor-Related Protein 1) amyloid-β receptors, in brain microvessels, suggesting a role in amyloid-β dysregulation (67). In addition, *in vitro* exposure of human cerebral microvascular endothelial cells to Tat also resulted in reduced expression of occludin and LRP1 while increasing RAGE expression without affecting cell viability (91). This dysregulation of LRP1 and RAGE expression (67) and BBB leakage (67, 90) by Tat gets mitigated with Rho Kinase inhibitor Hydroxyfasudil, suggesting involvement of Rho/Rock signaling in HIV-associated neurocognitive disorders. Additionally, Tat also interferes with neprilysin (NEP), a key enzyme expressed in cerebral microvascular endothelial cells, neurons, and astrocytes involved in amyloid-β metabolism (67).

HIV-Tat-mediated oxidative stress, which is involved in BBB injury, has also been associated with the development of depression-like behavioral changes in experimental models (92). Furthermore, doxycycline inducible expression of Tat in astrocytes resulted in oxidative stress and depression like behavior in GT-tg bigenic mice (93). A study by Lawson et al. found increased proinflammatory cytokine expression in the hippocampus and frontal cortex, brain regions commonly associated with depression in mice injected with Tat intracerebroventricularly (94) (**Table 1**).

**Table 1:** Molecular mechanisms of HIV-Tat-induced endothelial dysfunction across organ systems.

Organ System	Molecular Target	Pathological Outcome	Experimental Evidence	Ref
Brain (CNS)	ZO-1, Occludin, Claudin-5	Tight junction disassembly, BBB integrity disruption	Tat-injected WT mice, Evans Blue, and FITC-dextran assays	(67, 84, 85)
	VEGFR2	Disruption of tight junctions	Hippocampal Tat injection in WT mice, Rho/ROCK signaling-dependent activation	(86)
	GRP78, PERK, ATF6	ER stress and apoptosis	*In vitro* analysis of Tat-treated HBMECs	(88)
	Bcl2/Bax, Cytochrome c	Mitochondrial dysfunction and apoptosis	*In vitro* analysis of Tat-treated HBMECs	(88)
	RAGE, LRP1	Amyloid-β accumulation and dysregulation	Microvessel analysis of brains from Tat-administered WT mice	(67, 90, 91)
	ICAM-1	Increased cell adhesion and BBB inflammation	Tat-treated HBMECs and WT mouse microvessels	(52)
	MCP-1	Chemotaxis and neuroinflammation	Tat-treated astrocytes and HBMECs, hippocampal Tat injection in WT mice	(82, 165)
	E-selectin, IL-6	Endothelial activation and inflammation	*In vitro* analysis of Tat-treated HBMECs	(83)
	CREB	Regulates ZO-1 localization, endothelial barrier disruption	*In vitro* analysis of Tat-treated HBMECs	(86)
Cardiovascular System	VEGFR2/KDR	Cytoskeletal remodeling, pro-angiogenic activity	*In vitro*, Tat-treated human aortic endothelial cells	(99)
	αvβ3 Integrin	NF-κB activation, adhesion, proliferation	*In vitro*, Tat-treated endothelial cells	(100)
	eNOS	Depletion of nitric oxide, impairing vasorelaxation	TAT-treated porcine coronary arteries	(45)
	NOX1/NOXA1	ROS accumulation and vascular dysfunction	Tat-treated C57BL/6 mouse aortic tissue	(102)
	NF-κB	Pro-inflammatory gene expression	*In vitro* Tat-treated PAECs, HUVECs, and HBMECs; *in vivo*, Tat-treated WT mice	(50, 54, 55)
	PPARγ	Supression of vMF and VEGFR-1, VEGFR-2 endothelial markers	*In vitro*, Tat-treated mesenchymal stem cells	(109)
	miR-34a, miR-146a	Endothelial senescence	Tat-treated endothelial cells	(110)
Pulmonary System	PAK1	Cytoskeleton rearrangement	Tat-treated HLMVECs	(112)
	VCAM-1	Increased oxidative stress and inflammatory activation	*In vitro*, Tat-treated PAECS, *in vivo* SP-C Tat-transgenic mice	(55)
	MCP-1	Monocyte transmigration	*In vitro*, Tat-treated HLMVECs	(60)
	Caspase-3	Endothelial apoptosis	*In vitro*, Tat-treated PAECs	(113)
	Tip60	Inhibition of DNA damage response	*In vitro*, Tat-treated PAEC cells	(115)
	SOD2	Altered oxidative stress signaling	*In vitro*, PAECs and *in vivo* SP-C transgenic mice	(118)

## HIV-Tat and cardiovascular dysfunction

Cardiovascular dysfunction (CVD) remains a significant comorbidity among PLWH in the ART era and has become the leading cause of morbidity and mortality in these individuals (95). PLWH have an increased risk of developing hypertension, myocardial infarction, and atherosclerotic lesions. In addition, markers of subclinical atherosclerosis, such as increased carotid artery intima-media thickness, arterial stiffness, and reduced flow-mediated dilation, are also observed (96). The vascular endothelium is a key regulator of these processes, contributing to changes by modulating vascular tone, controlling blood flow, and coordinating inflammatory responses (97, 98). HIV-Tat, on binding to the vascular endothelial growth factor receptor-2/Kinase insert domain receptor (VEGFR2/KDR), can alter aortic endothelial cell behavior by modulating cytoskeletal organization and promoting pro-angiogenic activity (99). HIV-Tat also interacts with αvβ3 integrin of endothelial cells, thereby enhancing adhesion and proliferation of endothelial cells, and neovascularization by mediating downstream activation of focal adhesion kinase and nuclear factor-κB (NF-κB) signaling (100).

A study by Kress et al. demonstrated the stimulation of systemic hypertension and endothelial dysfunction following the transfer of CD4+ T cells from HIV transgenic mice expressing Tat and other proteins to wild-type mice. The viral proteins, including Tat, induced hypertension through IL-1α-mediated increases in NADPH oxidase 1 (NOX1) as well as subsequent increased Reactive Oxygen Species (ROS) and impaired vasodilation (101). Paladugu et al. further demonstrated the link between HIV-Tat and endothelial dysfunction via wire myography on porcine coronary artery rings. Tat exposure impaired endothelium-dependent vasorelaxation of porcine artery rings in response to bradykinin, which could be prevented in the presence of Tat neutralizing antibodies (45). Another study by Kovacs et al. used wire myography of thoracic aortic rings from Tat-transgenic mice, demonstrating that Tat contributed to reduced endothelium vasorelaxation (102). Intracellular Ca²^+^ plays a key role in endothelial and cardiovascular dysfunction. Tat contributes to cardiac dysfunction by elevating intracellular calcium levels in AC16 cardiomyocytes and modifying markers of endothelial toxicity (69). Tat-induced Ca²^+^ elevation occurs through lysosomal mobilization, endoplasmic reticulum release, and via Ca²^+^ influx through Transient Receptor Potential Vanilloid 2 (TRPV2) cation channel in cardiac parasympathetic neurons (103). Microinjections of Tat into the nucleus ambiguus of rats led to dose-dependent bradycardia, driven by neuronal TRPV2 activity (103). Given that the electrophysiological activity of cardiomyocytes depends on mitochondrial function, exposure of Tat to primary rat cardiomyocytes was reported to disrupt mitochondrial Ca²^+^ uptake, impair oxidative phosphorylation, with a reduction in ATP levels, and an increase in ROS accumulation (104).

Dilated cardiomyopathy has been observed in HIV-transgenic mice, expressing Tat predominantly in the heart muscle (105). These mice exhibited symptoms like reduced peak left ventricular systolic pressure (LVSP), increased left ventricular end-diastolic pressure (LVEDP), reduced contractility, and impaired diastolic relaxation (105). Global expressions of Tat in transgenic mice made the heart more vulnerable to endotoxin-induced injury, but Tat expression itself didn’t result in cardiac dysfunction, suggesting Tat sensitizes the heart to stress without independently affecting baseline cardiac performance (106). However, targeted expression of HIV Tat in mouse heart cells led to cardiomyopathy, marked by increased left ventricular mass, reduced heart function, elevated atrial natriuretic factor (ANF) mRNA, mitochondrial structural damage, and glutathione depletion (107). In another study, Tat expression in ventricular tissues of mice was found to be associated with increased levels in RAGE and SOD-2, along with cellular changes such as increased mast cells and collagen accumulation. However, echocardiographic analysis detected no differences in diastolic and systolic function between 2-6 month old Tat transgenic and Tat-negative wildtype animals in this study (69).

Atherosclerosis is one of the main cardiovascular disorders associated with HIV-Tat, and endothelial dysfunction represents an early step in the pathogenesis of atherosclerosis. Tat depletes NO by reducing both endothelial nitric oxide synthase (eNOS) expression (**Figure 1**) and NO production, thereby impairing endothelium-dependent vasorelaxation (45). Tat-induced ROS generation, through the activation of NADPH oxidases (e.g., NOX1, NOX2, NOX4) in the aortas of Tat-treated mice, showing elevated levels of NOX-1 and its coactivator NADPH oxidase 1 (NOXA1) (102). The ROS can further react with NO to form peroxynitrite, a damaging reactive nitrogen species that can further disrupt endothelial function (108). These processes, linked to the activation of transcription factor NF-kB, promote expression of adhesion molecules in human pulmonary artery endothelial cells, leukocyte adhesion, and trans-endothelial migration (50, 54, 55). This leads to an inflammatory vascular environment crucial for the initiation and progression of atherosclerosis. In HIV-Transgenic mice, expression of HIV proteins, including Tat, led to arterial stiffness and increased carotid intima-media thickness (cIMT), both clinical markers of atherosclerosis (96). In addition, research suggests that the HIV-Tat protein impacts on the survival and differentiation of mesenchymal stem cells in the vasculature could enhance the formation of atherosclerotic lesions (109). Tat exposure to these stem cells was observed to promotes their differentiation toward adipogenesis by activating Peroxisome Proliferator-Activated Receptor Gamma (PPARγ) and inhibit their differentiation to endothelial cells by suppressing the expression of VEGF-induced endothelial markers such as von Willebrand factor (vWF), Fms-like tyrosine kinase 1 (Flt-1), and Kinase insert domain receptor (KDR), also known as Vascular Endothelial Growth Factor Receptor 1 (VEGFR-1) and Vascular Endothelial Growth Factor Receptor 2 (VEGFR-2) respectively (**Figure 1**) (109).

In another study, HIV-Tat exposure significantly increased endothelial cell senescence by upregulating miR-34a and downregulating miR-146a, which could promote the vascular infiltration of immune cells and the development of atherosclerotic vascular disease (110). A synergistic effect between HIV-Tat and pro-atherogenic shear stress in aortic endothelial cells has also been demonstrated. This interaction enhances endothelial expression of the potent protease cathepsin K, known to remodel extracellular matrix and promote vascular remodeling, and thereby potentially augments the CVD observed in PLWH (43) (**Table 1**).

## HIV Tat and pulmonary vascular injury

The endothelial dysfunction plays a pivotal role in the development of PH disease, contributing to abnormal cell proliferation and neo-angiogenesis. These changes result in the formation of advanced plexiform lesions, a hallmark of PH pathology (111). HIV- Tat’s extracellular effects have also been implicated in pulmonary vascular dysfunction. Wu et al. (2004) demonstrated that HIV-Tat can induce significant angiogenic effects using human lung microvascular endothelial cells (HLMVEC), which could lead to vasculopathy conditions in AIDS patients. They found that Tat exposure led to actin cytoskeletal rearrangement in lung endothelial cells, promoting stress fiber disassembly and ruffle formation. This cytoskeletal rearrangement primarily occurred through the activation of p21-activated kinase 1(PAK1), c-Jun-N-terminal kinase and NADPH oxidase (112). Kai Liu and colleagues (2005) explored further the consequences of Tat exposure on human pulmonary arterial endothelial cells. Their findings reveal that Tat’s interaction with these cells escalates the oxidative stress and NF-kB activation-dependent expression of VCAM-1, which is a critical mediator in the development of pulmonary vasculopathy (55). Further the HIV-1 Tat can function as a proto-cytokine by triggering the PKC activation-dependent release of MCP-1 by human lung microvascular endothelial cells, which in turn promotes transmigration of monocytes across the endothelial monolayer (60).

HIV-1 Tat triggers apoptosis in lung microvascular endothelium by activating caspase-3 via a mechanism independent from the Fas pathway or TNF production (113). Alternatively, Tat has been reported to mediate a pro-survival cellular phenotype2 (114) by preventing caspase-mediated apoptosis in response to DNA damage (115). HIV-Tat is known to interact with histone acetyl transferase Tip60 (Tat-interacting protein 60 kDa), an important player in the DNA Damage Response. This interaction with Tat results in the inhibition of Tip 60 activity and its ability to respond to DNA damage and promote cell apoptosis (**Figure 1**) (115). In addition, Tat has been demonstrated to regulate the endothelial cell proliferation by binding α5β1/αvβ3 integrins via its arginine-glycine-aspartic (RGD) region and triggering Ras and ERK signaling (116).

Research on transgenic mice expressing the HIV-Tat highlighted the role of Tat in enhancing oxidative stress within the lung tissues. In these lung tissues, increased NF-κB activation, elevated levels of nitrotyrosine and thioredoxin interacting protein (TxNIP) with reduced levels of manganese superoxide dismutase (MnSOD)were seen as compared to wild-type mice, which indicated oxidative burden triggered by Tat in the pulmonary settings (117). Alternatively, *in vitro* analysis found Tat to promote the expression of Superoxide Dismutase 2 (SOD2) in pulmonary artery endothelial cells by influencing the SP1 and SP3 expression and the binding of Sp3 transcription factors to the SOD2 promoter regions. However, no change in the SOD2 expression was observed in the lung homogenates from HIV-infected humanized NSG-BLT Mice (118) (**Table 1**).

## Dual-hit of HIV-Tat and exposomes in vascular dysfunction

Deaths due to overdose on drugs of abuse reached a record high of 70,630 in 2019 in United States, and this is a particular problem in people living with HIV (119). According to CDC data, about 1 in 10 new HIV infections occurs in a person who injects illicit drugs (120). Among PLWH IDUs, 62% inject heroin daily, 54% use speedball (heroin and cocaine), and 35% inject methamphetamine (121). Illicit drug use contributes to a higher likelihood of engaging in unprotected sex, thereby increasing the risk of HIV transmission. Stimulant use is prevalent among sexual and gender minorities, and it has been linked to elevated risks of HIV acquisition, CVD-related mortality (122).

### Dual hit and brain dysfunction

HAND is of particular concern among HIV-infected individuals using illicit drugs (123–125). A study conducted between 2018 to 2019 in Baltimore reported a significant association of both HIV infection and female sex with neurocognitive impairment among cocaine users, suggesting that cocaine use may exacerbate HIV-related cognitive decline (124). HIV and substance use also impact brain regions linked to procedural memory. In a study involving abstinent individuals with a history of cocaine or heroin use, PLWH showed poorer performance on motor-based tasks, although their learning rates were comparable to HIV-negative individuals (126). Both HIV-infection and illicit drugs disrupt dopamine absorption and release by altering dopamine transporter function, leading to elevated extracellular dopamine levels, which can impact lymphoid, myeloid, and glial cell behavior (127). For instance, dopamine promotes the migration of CD14+CD16+ monocytes across the BBB, a critical concern since these monocytes harbor high levels of HIV DNA and are associated with cognitive impairment in people with HIV (128).

HIV, along with commonly abused substances such as cocaine, methamphetamine, alcohol, tobacco, opioids, and cannabinoids, synergistically disrupts the blood-brain barrier (BBB), intensifying neuroinflammation and accelerating the progression of HAND (129). Cocaine specifically increases BBB permeability in human brain endothelial cells by disrupting tight junctions and cytoskeletal integrity, while also enhancing CCL2/CCR2 signaling in monocytes, thereby worsening HIV-related neuroinflammation and neuropathogenesis (130). Additionally, cocaine use in PLWH upregulates expression of activated leukocyte cell adhesion molecule in brain endothelium, promoting monocyte adhesion and transmigration across the BBB (131). HIV-1 clade B Tat protein disrupts the blood-brain barrier (BBB) more significantly than clade C, with cocaine exacerbating this effect in a clade-specific manner. This disruption is linked to changes in tight junction protein expression, particularly ZO-1 and JAM-2 (132). Cocaine also enhances platelet–monocyte complexes, which may cross the BBB, and together with HIV proteins, activates JNK, p38, ERK/MAPK, and NF-κB pathways, leading to neuronal stress and the development of HAND (133). Studies have shown that both HIV infection and methamphetamine consumption increase fractional anisotropy, reflecting white matter tract disruption linked to cognitive decline (134). Furthermore, their combined negative effects on cerebral blood flow and functional blood flow regulation have been documented (135). In brain tissues, it has been demonstrated that HIV and methamphetamine use together contribute to global DNA methylation changes in genes associated with neurodegeneration, dopamine metabolism, transport, and oxidative phosphorylation, all of which are linked to neuropsychiatric disorders (136). HIV-Tat and methamphetamine synergistically disrupt blood-brain barrier (BBB) integrity through multiple mechanisms. Synergistically, they impair transcellular transport of therapeutic drugs by suppressing P-glycoprotein (P-gp) function and multidrug resistance protein 1 (MRP-1) (137). Further, this combination increases oxidative stress via transient receptor potential melastatin 2 (TRPM2) channel activation leading to tight junction protein loss (JAMA, Occludin, ZO1), apoptosis, and BBB leakage (**Figure 1**) (138). In neuron-astrocyte cultures, Tat and methamphetamine enhance MMP-1/2 and urokinase plasminogen activator (uPA) via Gi/Go signaling, exacerbating neuroinflammation and BBB damage (139).

Additionally, both *in vitro* and *in vivo* studies demonstrated Tat and methamphetamine mediated synergistic downregulation in the expression of glucose receptors and tight junction proteins and an increase in oxidative stress and BBB permeability (140, 141). Another HIV-1 Tat-transgenic mouse model study revealed that fentanyl abuse alone significantly disrupts BBB integrity by reducing tight junction proteins and altering VCAM and PDGFR-β expression. Fentanyl also dysregulated immune responses, with strong associations between inflammatory markers and BBB disruption. These findings highlight the neurotoxic potential of fentanyl and its synergistic risk in the context of HIV (142). Finally, in recent studies, showing significantly higher risk of brain white matter hyperintensities (WMH) in PLWH compared to HIV-negative controls was found to be linked to tobacco use (143, 144).

A study by Nass et al. demonstrated that Tat and morphine exposure in mice produced depressive-like behaviors and contributed to dendritic spine loss in the prefrontal cortex, a region associated with mood regulation. The combined use of Tat and morphine exacerbated neuronal injury and promoted the dysregulation of microglial response to immune stimulation, indicating innate immune fatigue (145). However, the direct pathological interactions of Tat and morphine on the cerebrovascular endothelium may also contribute to the development of depression observed in PLWH.

### Dual hit and cardio-pulmonary dysfunction

Similar to HIV associated brain dysfunction, illicit drugs such as cocaine, heroin, morphine, and methamphetamines are strongly linked to HIV associated cardio-pulmonary complications, with cocaine and methamphetamine identified as independent risk factors for PH development, even after adjusting for other contributing conditions (146–148). In a French study, HIV-PAH prevalence was 8.2%, with injection drug use (IVDU) as the leading risk factor, especially in 57% of severe PAH (NYHA stage IV) cases. 19770696 Recent registry data show HIV-PAH in 1.43% of patients, marked by higher heart rate and pulmonary resistance. Methamphetamine use was notably higher in HIV-PAH cases (36%) than in idiopathic PAH (6%) (149).

Our group reported disruption of tight junction protein ZO-1 in human pulmonary microvascular endothelial cells on the combined exposure to HIV-Tat and cocaine, leading to an additive increase in the endothelial permeability (**Table 2**). This disruption was found to be mediated through oxidative stress and activation of Ras/ERK1/2 signaling pathway. Pre-treatment with SU5416 (VEGFR-1 antagonist), BD1047 (sigma receptor antagonist) or NADPH oxidase inhibitor significantly attenuated the Tat and cocaine mediated endothelial dysfunction (150). A synergy between HIV-Tat and morphine in mediating pulmonary vascular endothelial dysfunction has also been reported (**Table 2**). Heroin (diacetylmorphine), which is biochemically converted to morphine when consumed, could also lead to similar adverse effects as morphine (151, 152). Rhesus macaques infected with SIVmacR71/17E and treated with morphine exhibited significantly higher pulmonary vascular remodeling, including early and advanced plexiform lesions, compared to SIV-only or morphine-only treated macaque controls (153). Enhanced oxidative stress was found to increase endothelial cell apoptosis, followed by compensatory proliferation on the combined exposure of Tat and drug exposure, including morphine, cocaine or methamphetamine, than with either condition alone (153). A follow-up study by Dalvi et al. reported involvement of maladaptive autophagy in shifting early apoptotic endothelial cells to later apoptotic-resistant proliferative endothelial cells in response to Tat and morphine. Oxidative stress was found to be playing a role in triggering the autophagic pathway (154). Continuing with this, our team further identified the role of NADPH oxidases (NOX) in Tat and morphine mediated oxidative stress in pulmonary microvascular endothelial cells. Enhanced activity of NOX2 and NOX4 isoforms was found to be the primary source of oxidative stress, and this was associated with pulmonary vascular remodeling and increased right ventricular systolic pressure in HIV-transgenic rats treated with morphine (114) (**Table 2**).

**Table 2:** Synergistic effects of HIV-Tat and illicit drugs on endothelial dysfunction.

Drug of Abuse	Synergistic Mechanism with HIV-Tat	Endothelial Consequences	Organ-Specific Impact	Citation
Cocaine	Ras/ERK1/2 pathway and ROS production	Loss of ZO-1, endothelial permeability, activation of pro-inflammatory signaling	Pulmonary Vascular Injury	(150)
	Increases platelet–monocyte complexes, co-activates JNK, ERK/MAPK, and NF-κB	Increased BBB permeability, ZO-1 and JAM-2 disruption, neuronal stress	BBB disruption, development of HAND	(132, 133)
Methamphetamine (Meth)	Impaired therapeutic drug transport by suppression of P-glycoprotein integrity and MRP-1 in HPMECs	ZO-1 and Occludin disruptions and decreases in BBB drug efflux	Endothelial dysfunction in brain vasculature, neurotoxicity	(137)
	Increased ROS through TRPM2 channel activation	ZO-1 loss, Endothelial apoptosis, BBB leakage	Neuronal inflammation, inflammatory cell migration, CNS damage	(138)
	Enhance MMP-1/2 and uPA via Gi/Go signaling in neuron-astrocyte cultures	Endothelial permeability and BBB damage	Neuronal inflammation, monocyte transmigration, and BBB damage	(139)
	Downregulation of GLUT1, GLUT3, and increased ROS	Loss of tight junction proteins and transporter dysfunction, BBB leakage	BBB disruption, ultrastructural damage to the brain	(140, 141)
		Endothelial apoptosis on acute exposure, chronic exposure leads to increased proliferation	Pulmonary vascular remodeling	(153)
Morphine	Increased ROS, alteration in VEGFR-2 activation/expression	Endothelial apoptosis followed by enhanced proliferation	pulmonary vascular remodeling, angio-obliteration, plexiform lesion formation in SIV-infected macaques	(153)
	Increased ROS, upregulation of ULK1, Beclin-1, ATG5, ATG7 in PME cells	Increased autophagy, increased proliferative phenotype	Augmentation of vascular remodeling	(154)
	Co-activation NOX2 and NOX4, increased oxidative stress	Increased inflammation, increased endothelial permeability	Pulmonary vascular remodeling, elevated right ventricular systolic pressure in rat model.	(114)
Fentanyl	Altered VCAM and PDGFR-β expression	Disrupted tight junctions, enhanced neuroinflammation	Increased neurotoxicity, BBB disruption	(142)

Furthermore, in a cohort of 74 polysubstance-using women living with HIV, elevated NT-proBNP (N-terminal pro-B-type natriuretic peptide) levels, a marker of cardiac stress, were positively associated with sTNFR2 (soluble tumor necrosis factor receptor 2), suggesting a link between inflammation and cardiac dysfunction in this population (155). Additionally, menthol cigarette smokers among PLWH had twice the risk of hypertension, greater BMI, and abdominal obesity compared to non-smokers, along with a twofold higher likelihood of moderate to high cardiovascular risk (156). Hazardous drinking and alcohol abuse were also significantly associated with increased CVD risk in PLWH men, even after adjusting for traditional and HIV-specific risk factors, indicating an independent contribution of alcohol to cardiovascular complications (157). However, studies demonstrating the combinatory effects of HIV-Tat and alcohol/cigarette smoke on vascular endothelium are warranted to understand the CVD risk in HIV infected cigarette smokers or alcohol abusers.

## Tat–targeted therapeutic approaches

Targeting the interactions between HIV-Tat and TAR, as well as Tat and host cellular proteins, represents a crucial therapeutic strategy in HIV treatment. These interactions are essential for efficient viral transcription and replication, and their inhibition offers a promising approach to suppress viral propagation and potentially induce or maintain latency. In a small-molecule microarray screen, a 6-ethyl-5-methylthienopyridine derivative was identified that selectively binds the HIV-TAR RNA hairpin, resulting in reduced viral replication in CEM-SS cells (158). Similarly, a fragment-based drug design strategy led to the identification of an indole tetrahydropyrimidine compound as a potential Tat–TAR binding inhibitor (159). The development of small molecules that mimic HIV-Tat protein has been extensively explored, particularly through peptidomimetic strategies aimed at enhancing TAR-binding affinity. Among these, tyrosine oligomers and their derivatives demonstrated effective inhibition of the Tat–TAR interaction in peripheral blood mononuclear cells (PBMCs) (160). Among all known Tat inhibitors, didehydro-Cortistatin A (dCA) remains one of the most promising, as it directly binds to Tat and disrupts its function (161). Chromatin immunoprecipitation studies have shown that dCA not only blocks RNA polymerase II-mediated transcriptional elongation from the 5′ LTR but also inhibits transcriptional initiation in chronically infected cells. This blockade results in a multi-fold decrease in both viral mRNA and viral particle production (162). Another noteworthy Tat-mediated transcription inhibitor (TMTI) is the diterpenoid epoxide triptolide, which interferes with Tat activity by promoting its degradation. This effect has been validated in both Jurkat T cells and PBMCs (163). In addition to Tat–TAR disruption, protein–protein interactions (PPIs) between Tat and host cellular factors are also critical for HIV replication. One such interaction is with protein phosphatase 1 (PP1) that promotes Tat induced HIV transcription. PP1 is a serine/threonine phosphatase that dephosphorylates the host cell transcription factor CDK9/cyclin T1 at Thr186, thereby enhancing viral transcription. An acridine-based compound, 1H4, which mimics the action of the central domain of the nuclear inhibitor of PP1 (cdNIPP1), was found to effectively inhibit HIV-1 transcription and viral replication at non-cytotoxic concentrations in MT-4 cells (164). Although these findings demonstrate Tat inhibition and associated reduction in viral replication, their implications in HIV-induced endothelial dysfunction remain unclear. Restoration of endothelial integrity, especially in vital organs, is critical. Therefore, further research is needed to address these existing knowledge gaps and to improve the therapeutics.

## Conclusion

In summary, despite the success of antiretroviral therapy in controlling viral replication, HIV-Tat persists and continues to play a significant role in promoting brain vascular injury, neuroinflammation, cardiovascular dysfunction, and pulmonary vascular injury. The common pathogenic mechanism behind these complications mainly includes endothelial dysfunction and loss of vascular integrity. Tat effect on endothelium is highly complex and multifaceted that primarily includes oxidative stress, activation of inflammatory pathways, disruption of endothelial tight junctions, increased cell adhesion, and mitochondrial dysfunction (**Figure 1**). It promotes vascular proinflammation by activating the TLR4-NFκβ signaling pathway along with increased expression of adhesion molecules, including RAGE, ICAM, and VCAM. Extracellular Tat also enhances endothelial cell proliferation through upregulation of the VEGF-A/VEGFR-2 signaling cascade, the HIF-1α/PDGF-BB axis, and activation of Rho, Ras, and Notch signaling pathways, while concurrently suppressing the DNA damage response. Additionally, eTat exacerbates oxidative stress by increasing the expression of eNOS and SOD2. It further compromises endothelial barrier integrity by disrupting tight junctions, characterized by reduced levels of ZO-1, occludin, and claudin-5, and by enhancing MMPs through Rho and Ras pathway activation (**Figure 1**). In addition, HIV-Tat has been proven to act synergistically with drugs of abuse in mediating vascular damage and exacerbating brain, lung, and heart disease progression. Given that ART alone cannot completely aid in the full recovery of PWH, targeted interventions to prevent the chronic deleterious effects of HIV-Tat on the vasculature are strongly recommended. Further research is essential to fully elucidate the molecular mechanisms of Tat-induced vascular injury and to identify potential therapeutic strategies. The development of small molecules or immunologically active principles capable of mitigating HIV-Tat-induced vascular damage holds great promise for improving clinical outcomes in individuals living with HIV.

## Glossary

HAART: Highly Active Antiretroviral Therapy
ART: Antiretroviral Therapy
Tat: Trans activator of Transcription
PLWH: People living with HIV
CVD: Cardiovascular Disease
PAH: Pulmonary Arterial Hypertension
PH: Pulmonary Hypertension
PH-LHD: Pulmonary Hypertension with left heart disease
cART: Combination Antiretroviral Therapy
HAND: HIV-associated neurocognitive disorder
LTR: Long Terminal Repeat
TAR: Transactivation Response Region
NF-κB: Nuclear Factor kappa B
TRAF6: Tumor Necrosis Factor Receptor-Associated Receptor 6
SIV: Simian Immunodeficiency Virus
CNS: Central Nervous System
ECs: Endothelial Cells
VCAM-1: Vascular Cell Adhesion Molecule 1
ECLAM-1: Endothelial Leucocyte Adhesion Molecule-1
ICAM-1: Intracellular Adhesion Molecule-1
TLR4: Toll-like Receptor 4
PKC: Protein Kinase C
MAP: Mitogen-Activated Protein Kinase
TNFα: Tumor Necrosis Factor-alpha
IL-10: Interleukin-10
IL-1β: Interleukin-1 beta
IL-6: Interleukin-6
MCP-1: Monocyte Chemoattractant Protein-1
BBB: Blood Brain Barrier
HBMECs: Human Brain Microvascular Endothelial Cells
CCL-2: C-C Motif Chemokine Ligand 2
TJs: Tight Junctions
Zo-1: Zonula Occludens-1
VEGFR1: Vascular Endothelial
VEGFR2: Vascular Endothelial Growth Factor Receptor 2
CREB: Cyclic AMP Response Element-Binding Protein
PPARγ: Peroxisome Proliferator-Activated Receptor
Bcl2: B-cell lymphoma 2
Bax: Bcl-2-associated X protein
MMP-9: Matrix Metalloproteinase-9
RAGE: Receptor for Advanced Glycation End Products
LRP1: Low-Density Lipoprotein Receptor-Related Protein 1
TRPV2: Transient Receptor Potential Vanilloid 2
ROS: Reactive Oxygen Species
SOD2: Superoxide Dismutase 2
NO: Nitric Oxide
NOXA1: NADPH oxidase 1
KDR: Kinase Insert Domain Receptor
HLMVEC: Human Lung Microvascular Endothelial Cells
PAK1: p21-activated kinase 1
Tip60: Tat interacting Protein 60 kDa
RGD: Arginine-Glycine-Aspartic
MnSOD: Manganese Superoxide Dismutase
JAM-2: Junctional Adhesion Molecule 2
WHO: World Health Organization
TRIF: TIR-domain-containing adapter-inducing interferon-beta
PASP: Pulmonary Artery Systolic Pressure
NSG-BLT: NOD-scid IL2Rγnull Bone Marrow-Liver-Thymus Humanized Mice
JNK: c-Jun N-terminal kinase
PDGFR-β: Platelet-Derived Growth Factor Receptor beta
PLWH: People Living with HIV.
